# METABOLOMICS PROFILING OF MUSCLE FAT DEPOSITION IN AGING: RESULTS FROM THE HEALTH ABC STUDY

**DOI:** 10.1093/geroni/igz038.3457

**Published:** 2019-11-08

**Authors:** Samaneh Farsijani, Megan M Marron, Iva Miljkovic, Mary E Baugh, Stephen Kritchevsky, Anne B Newman

**Affiliations:** 1 University of Pittsburgh, Pittsburgh, Pennsylvania, United States; 2 Wake Forest School of Medicine, Winston-Salem, United States; 3 Wake Forest School of Medicine, Winston-Salem, North Carolina, United States

## Abstract

Background: Age-related inter-muscular fat (IMF) deposition is associated with poor physical function in those with preserved/high muscle mass. However, the heterogeneity of IMF in aging is poorly understood. We used a semi-targeted metabolomics approach to: 1) determine the metabolites associated with IMF infiltration in aging; and 2) establish a model to predict IMF using the metabolome. Methods: We performed a cross-sectional analysis of 314 African-American men (age: 69-79 years) from the Health ABC study at baseline. Mid-thigh IMF area (cm2, by CT) and 350 plasma metabolites (by liquid-chromatography/mass spectrometry) were measured. Correlation analysis was performed to determine metabolites associated with IMF. An IMF prediction model was calculated, using regression analysis with 10-fold cross-validations on random halves of the population with metabolites, age and weight as predictors. Results: Of 161 metabolites correlated with IMF (P<0.05), 34 remained significant after adjusting for age, weight, physical activity, medications, smoking and multiple comparisons (false discovery rate ≤0.25). IMF-associated metabolites were primarily lipids (76%) and amino acids (15%). Most metabolites were positively correlated with IMF (94%), with the exception of mevalonic acid (from fatty acids sub-class) and glutamine (from organic-acids) which were negatively associated with IMF. IMF-associated metabolites predicted 49% of the variability in the actual IMF in the test set of the random half of the population (50%, n= 144). Conclusion: Identification of the unique metabolomics features associated with IMF may improve our understanding of key biological alterations of muscle during aging.

